# Anterior chamber flare and ciliochoroidal detachment using flare photometry and anterior segment optical coherence tomography in acute lupus choroidopathy: A case report

**DOI:** 10.1016/j.ajoc.2022.101314

**Published:** 2022-01-31

**Authors:** Satoko Fujimoto, Taku Wakabayashi, Kazuichi Maruyama, Chikako Hara, Eri Oguro-Igashira, Masayuki Nishide, Hirokazu Sakaguchi, Atsushi Kumanogoh, Kohji Nishida

**Affiliations:** a Department of Ophthalmology, Osaka University Graduate School of Medicine, 2-2 Yamadaoka, Suita, Osaka, 565-0871, Japan; b Department of Innovative Visual Science, Graduate School of Medicine, Osaka University, 2-2 Yamadaoka, Suita, Osaka, 565-0871, Japan; c Department of Respiratory Medicine and Clinical Immunology, Graduate School of Medicine, Osaka University, 2-2 Yamadaoka, Suita, Osaka, 565-0871, Japan

**Keywords:** Systemic lupus erythematosus, Lupus choroidopathy, Ciliochoroidal detachment, Anterior segment optical coherence tomography, Laser flare photometry, Flare meter

## Abstract

**Purpose:**

To report anterior chamber flare using laser flare photometry and ciliochoroidal detachment using anterior segment optical coherence tomography (AS-OCT) in a new onset acute lupus choroidopathy case.

**Observations:**

A 57-year-old woman with severe nephritis, pleural effusion, and ascites was referred to our ophthalmology clinic for rapid onset of bilateral blurred vision and eyelid swelling. She had a bilateral high-flared, shallow anterior chamber, and bilateral ciliochoroidal detachment, which was revealed using laser flare photometry and AS-OCT. She also had a serous retinal detachment and disc-macular retinoschisis with a thicker choroid and waved Bruch's membrane. Indocyanine green angiography (ICGA) demonstrated partial hypocyanescence in the early phase and multiple hypercyanescent spots at the intermediate to late phase, which are typical of lupus choroidopathy. Systemic lupus erythematosus was diagnosed, and after the administration of pulse methylprednisolone and pulse cyclophosphamide therapies, all eye findings completely resolved in a month, and all other signs and symptoms improved.

**Conclusions and Importance:**

Lupus choroidopathy, which is less common than retinopathy, might be under-diagnosed because of its difficult evaluation. Although ICGA is the gold standard for diagnosing lupus choroidopathy, a high flare of the anterior chamber and ciliochoroidal detachment might be different from lupus retinopathy. Laser flare photometry and AS-OCT can be non-invasive, helpful tools for the longitudinal evaluation of the patient's response to therapy.

## 1 Introduction

Systemic lupus erythematosus (SLE) is a chronic autoimmune disease affecting multiple organs. Its typical ocular manifestations include keratoconjunctivitis sicca, scleritis, retinopathy, and neuro-ophthalmic diseases.^1^ Lupus choroidopathy, which is a rare complication usually accompanied by severe renal and central nervous system disorders, has been possibly under-diagnosed because of its difficult evaluation.^1^, ^2^, ^3^ This study reports on the diagnosis of new-onset acute lupus choroidopathy using multimodal imaging. Non-invasive and longitudinal analysis of the anterior high flare and ciliochoroidal detachment by laser flare photometry and anterior segment optical coherence tomography (AS-OCT), respectively, was particularly useful for evaluating the degree of inflammation associated with lupus choroidopathy and subsequent response to therapy.

## 2 Case report

A 57-year-old Japanese woman, who suffered from Raynaud's phenomenon, and fatigue for two years, and had experienced a pituitary gland stroke four months earlier, was admitted to the hospital for worsening shortness of breath and abdominal distension. Fever, pleural effusion, and ascites were observed. Laboratory data revealed severe hypocomplementemia, hypoalbuminemia, severe proteinuria, hematuria, positive antinuclear antibodies, anti-DNA antibodies, and lupus anticoagulant. Following renal biopsy, lupus nephritis class IV-G (A) based on the International Society of Nephrology/Renal Pathology Society classification was diagnosed. The patient fulfilled the diagnostic criteria stated by the 2019 European League Against Rheumatism/American College of Rheumatology Classification Criteria for SLE.^4^ At the same time, the patient presented to our ophthalmology clinic with seven days of blurred vision, eyelid edema, and chemosis.

**Fig. 1:**
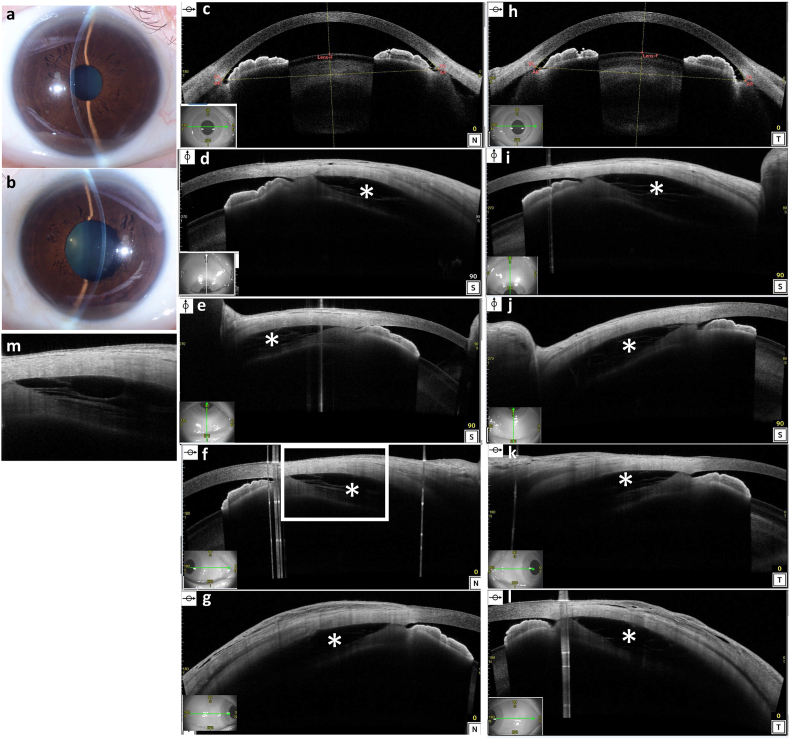
Anterior slit lamp photographs and anterior segment optical coherence tomography (AS-OCT) images of acute lupus choroidopathy. **(a, b)** the anterior slit lamp photographs showing shallow anterior chamber (right eye and left eye). **(c)** AS-OCT image of the shallow anterior chamber of the right eye. **(d**–**g)** AS-OCT images of the right eye showing quadrants (superior, inferior, nasal**,** and temporal of the ciliary body). **(h)** AS-OCT image of the shallow anterior chamber of the left eye. **(i**–**l)** AS-OCT images of the left eye showing quadrants (superior, inferior, nasal**,** and temporal of the ciliary body). Asterisks indicate the ciliochoroidal detachment. **(m)** magnified image of **1f**. The lamellar ciliary muscles, namely, ciliary-schisis could be detected in the detached area.

Ophthalmic examination revealed a best corrected visual acuity of 20/20 in both eyes, normal intraocular pressure (IOP), slightly higher left IOP (right, 13 mmHg; left, 21 mmHg), eyelid and conjunctival swelling, and shallow anterior chambers with neither hyperemia nor cells in the anterior chamber (Fig. 1a and b).

**Fig. 2:**
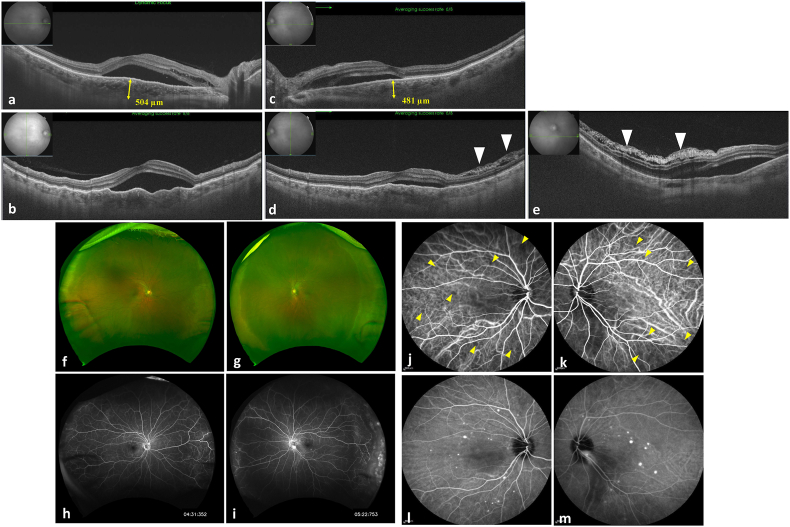
Posterior segment optical coherence tomography (PS-OCT) images, color photographs, fluorescence angiography (FA) images, and indocyanine green angiography (ICGA) images of acute lupus choroidopathy. **(a, b)** horizontal and vertical PS-OCT images of the right eye with subretinal fluid, retinoschisis, and thick choroid. The choroidal thickness at the fovea was 504 μm. **(c**–**e)** horizontal, vertical**,** and inferior-to the disc PS-OCT images of the left eye with subretinal fluid, retinoschisis, and thick choroid. The choroidal thickness at the fovea was 481 μm. On the left eye, Bruch's membrane undulation and cystic space at the lower part of the nerve fiber layer (white arrow heads) can be seen. **(f, g)** color photographs of the right eye and the left eye showing the temporal and nasal peripheral choroidal detachments. **(h)** intermediate-phase FA image of the right eye showing subtle leak points at the posterior pole. **(i)** intermediate-phase FA image of the left eye showing subtle leak points at the posterior pole and at disc. Peripheral choroidal detachment looks like diffuse capillary leakage, vascular anomalies or avascular area. **(j, k)** early-phase ICGA image of the right eye and the left eye showing partial hypocyanine choroidal circulation (yellow arrow heads). **(l, m)** late-phase ICGA image of the right eye showing multiple hypercyanescent spots in the macular area of both eyes and the inferior disc area of the left eye. (For interpretation of the references to color in this figure legend, the reader is referred to the Web version of this article.)

Laser flare meter (Laser flare meter FM-600; KOWA Company, Tokyo, Japan) showed a high flare of the anterior chamber: right, 33.2 ± 3 photon counts per millisecond (ph/ms) and left, 82.6 ± 16.3 ph/ms. AS-OCT (CASIA2; Tomey, Nagoya, Japan) showed ciliochoroidal detachment between the ciliary body and sclera (Fig. 1c–l) which was difficult to detect using slit lamp or fundus photos (Fig. 2f and g). AS-OCT detected the lamellar ciliary muscles, namely, ciliary-schisis in the detached area of the wide ciliochoroidal detachment (Fig. 1m).

In the fundus, the temporal and nasal peripheral choroidal detachment was detected in both of the eyes (Fig. 2f and g). Posterior segment OCT (PS-OCT) revealed serous retinal detachment, waved Bruch's membrane, and thicker choroid in the macular area of both eyes and the inferior-to-the disc area of the left eye (Fig. 2a–e). The choroidal thickness of the right eye at the fovea was 504 μm and that of the left was 481 μm. Indocyanine green angiography (ICGA) demonstrated partial hypocyanescence in the early phase (Fig. 2j and k) and multiple hypercyanescent spots in the macular area of both of the eyes and the inferior disc area of the left eye at intermediate to late phase (Fig. 2l, m). These findings are typical of lupus choroidopathy.^5^^,^^6^ Fluorescein angiography (FA) showed leakage at the peripheral choroidal detachment and multiple hyperfluorescent spots as demonstrated by ICGA (Fig. 2h and i).

**Fig. 3:**
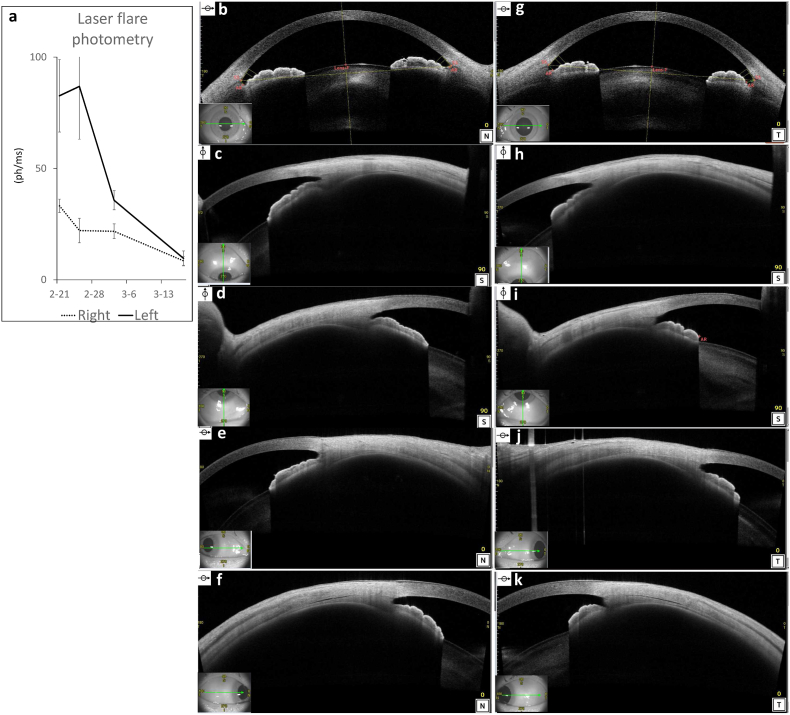
(a) The graph shows that the anterior chamber flare decreased as the treatment was administered. **(b)** anterior segment optical coherence tomography (AS-OCT) image of the right eye showed the normalized anterior chamber and no ciliochoroidal detachment. **(c**–**f)** AS-OCT images of the right eye showing quadrants (superior, inferior, nasal**,** and temporal of the ciliary body). **(g)** AS-OCT image of the left eye. **(h**–**k)** AS-OCT images of the left eye showing quadrants (superior, inferior, nasal**,** and temporal of the ciliary body).

**Fig. 4:**
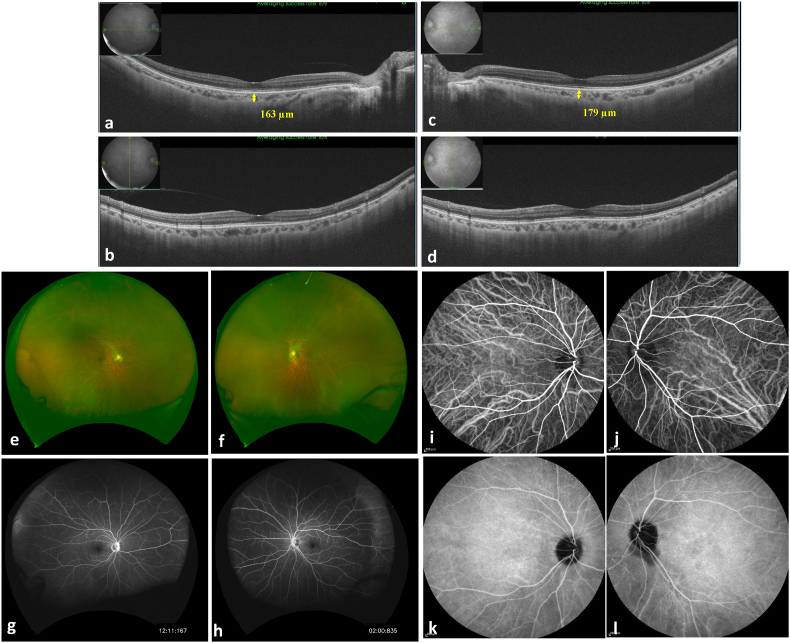
Posterior segment optical coherence tomography (PS-OCT) images, color photographs, fluorescence angiography (FA) images and indocyanine angiography (IA) images 3 months after acute lupus choroidopathy. **(a, b)** Horizontal and vertical PS-OCT images of the right eye. The choroidal thickness at the fovea was 163 μm. **(c, d)** Horizontal and vertical PS-OCT images of the left eye. The choroidal thickness at the fovea was 179 μm. **(e, f)** Color photographs of the right eye and the left eye. **(g, h)** Intermediate-phase FA images of the right eye and the left eye showing no leakage or hyper fluorescence spots. Peripheral findings also disappeared. **(i, j)** Early-phase IA images of the right eye and the left eye showing normal choroidal perfusion. **(k, l)** Late-phase IA images of the right eye and the left eye showing no hypercyanescent spots. (For interpretation of the references to color in this figure legend, the reader is referred to the Web version of this article.)

SLE was diagnosed, and pulse methylprednisolone therapy (1 g/day × 3 days) and pulse cyclophosphamide therapy (500 mg/body/2 weeks × 6) were administered, followed by oral administration of prednisolone at 60 mg/day. Her systemic findings gradually improved, and all eye findings completely resolved. The anterior flare decreased in a month as the treatment was administered (Fig. 3a), while ciliochoroidal detachment gradually disappeared in two months, and the shallow anterior chamber was normalized (Fig. 3b–k). It took two months for the retina to be completely attached and for choroidal thickness to be completely normalized to 163 μm (right) and 179 μm (left) (Fig. 4a–d). FA/ICGA also revealed that all leakage or hyperfluorescent spots disappeared after the therapy was administered (Fig. 4g–l).

## 3 Discussion

We encountered a case of acute lupus choroidopathy, which was diagnosed using common ICGA findings, such as choroidal hypoperfusion and focal hyperfluorescent spots^5^^,^^6^ We non-invasively evaluated anterior high flare and ciliochoroidal detachment with laser flare photometry and AS-OCT, respectively, which may not be checked in lupus retinopathy. Lupus choroidopathy is generally caused by immune complex deposition at the choroid, autoantibodies against retinal pigment epithelium, and thrombotic microangiography that cause tissue damage and organ dysfunction.^7^ The common ICGA findings in our case may represent immune complex depositions at the basement membrane of the choroidal vessels and Bruch's membrane.^8^^,^^9^ Our report showed concomitant ciliary detachment and choroidal detachment associated with acute lupus choroidopathy, which may support previous reports of secondary angle-closure glaucoma caused by lupus choroidopathy.^10^, ^11^, ^12^ AS-OCT enabled clear observation of the lamellar ciliary muscles, namely, ciliary-schisis in the detached area of the wide ciliochoroidal detachment in lupus choroidopathy. Choroidal detachment with a non-rhegmatogenous retinal detachment was defined as uveal effusion by Schepens^13^ in 1963, so our case could also be considered to present uveal effusion as reported in the previous studies.^10^, ^11^, ^12^ Uveal effusion is known to be caused by low intraocular pressure,^14^ malignant tumors,^15^^,^^16^ choroidal hypoperfusion owing to abnormally thick sclera^17^^,^^18^ or strong choroidal inflammation, such as that occurs in pan-retinal photocoagulation,^19^ Vogt-Koyanagi-Harada syndrome,^20^ and scleritis.^21^ Stefater et al.^22^ analyzed the suprachoroidal fluid of a patient with choroidal effusion and reported that the fluid was exudative, which indicates that the suprachoroidal fluid had a high protein concentration. Despite the absence of any visible anterior inflammation, our case showed a highly flared anterior chamber, which also indicates the high protein content of the aqueous humor. It seems contradictory whether anterior chamber flare rises in posterior uveitis^23^^,^^24^; however, it is reported that the anterior chamber flare level of associated blood-aqueous barrier disruption for reliable follow-up of posterior uveitis was empirically determined to be 13–15 ph/ms.^25^ Our case showed a higher flare than 15 ph/ms; flare rise in our case may have resulted from the breakdown of the blood-aqueous barrier due to the severe inflammation. Our result suggests that immune complexes may be deposited on the non-pigmented epithelium of the ciliary process or the endothelial cells of the iris, where the blood-aqueous barrier exists. In a mouse model of SLE, as well as the choroidal vessels and Bruch's membrane, immune globulin deposits were identified between the basement laminae of the endothelium and the pigmented epithelium of the ciliary process, accompanied by alterations in the epithelial basement membranes.^26^

Pulse methylprednisolone and cyclophosphamide therapy followed by oral prednisolone was effective in decreasing the anterior flare and re-attaching the ciliary body and the choroid. Non-invasive imaging using laser flare photometry and AS-OCT were not only useful for evaluating the degree of inflammation at the time of diagnosis but also for monitoring therapeutic efficacy in lupus choroidopathy.

## 4 Conclusions

In summary, we reported a case of acute lupus choroidopathy. Although FA/ICGA is the gold standard for diagnosing lupus choroidopathy, a high flare of the anterior chamber and ciliochoroidal detachment might be different from lupus retinopathy. Laser flare photometry and AS-OCT can be non-invasive, helpful tools for longitudinal evaluation of the patient's response to therapy.

## Patient consent

The patient provided written consent for publication of medical details and photographs.

## Funding

No funding was received for this work.

## Intellectual property

We confirm that we have given due consideration to the protection of intellectual property associated with this work and that there are no impediments to publication, including the timing of publication, with respect to intellectual property. In so doing we confirm that we have followed the regulations of our institutions concerning intellectual property.

## Research ethics

We further confirm that any aspect of the work covered in this manuscript that has involved human patients has been conducted with the ethical approval of all relevant bodies and that such approvals are acknowledged within the manuscript.

Written consent to publish potentially identifying information, such as details or the case and photographs, was obtained from the patient(s) or their legal guardian(s).

## Authorship

All listed authors meet the ICMJE criteria.  We attest that all authors contributed significantly to the creation of this manuscript, each having fulfilled criteria as established by the ICMJE.

We confirm that the manuscript has been read and approved by all named authors.

We confirm that the order of authors listed in the manuscript has been approved by all named authors.

## Author's contributions

SF and TW conducted research; SF, TW, KM, CH, HS, and KN wrote the manuscript; and EO, MN, and AK made the diagnosis and managed the systematic treatment.

## Declaration of competing interest

No conflict of interest exists.
